# Association of abnormal carbon dioxide levels with poor neurological outcomes in aneurysmal subarachnoid hemorrhage: a retrospective observational study

**DOI:** 10.1186/s40560-018-0353-1

**Published:** 2018-12-17

**Authors:** Shota Yokoyama, Toru Hifumi, Tomoya Okazaki, Takahisa Noma, Kenya Kawakita, Takashi Tamiya, Tetsuo Minamino, Yasuhiro Kuroda

**Affiliations:** 1 Department of Cardiorenal and Cerebrovascular Medicine, Kagawa University Hospital, 1750-1 Ikenobe, Miki, Kita, Kagawa 761-0793 Japan; 2 Department of Critical and Emergency Medicine, St. Luke’s International Hospital, 9-1 Akashi-cho, Chuo-ku, Tokyo, 104-8560 Japan; 3 Emergency Medical Center, Kagawa University Hospital, 1750-1 Ikenobe, Miki, Kita, Kagawa 761-0793 Japan; 4 Department of Neurosurgery, Kagawa University Hospital, 1750-1 Ikenobe, Miki, Kita, Kagawa 761-0793 Japan

**Keywords:** Subarachnoid hemorrhage, PaCO_2_, Hypocapnia, Hypercapnia, Intensive care unit, Neurological outcome, Delayed cerebral ischemia

## Abstract

**Background:**

In patients with aneurysmal subarachnoid hemorrhage (SAH), an association between hypocapnia and poor clinical outcomes has been reported. However, the optimal arterial carbon dioxide tension (PaCO_2_) remains unknown. The present retrospective study aimed to examine the association of abnormal PaCO_2_ levels with neurological outcomes and investigate the optimal target PaCO_2_ level in patients with SAH.

**Methods:**

We retrospectively selected consecutive adult patients hospitalized in the intensive care unit (ICU) for SAH between January 2009 and April 2017. Univariate and multivariate analyses were performed to identify the independent predictors of unfavorable neurological outcomes (i.e., modified Rankin scale score of 3–6 on hospital discharge).

**Results:**

Among 158 patients with SAH, 73 had unfavorable neurological outcomes. During the first 2 weeks in the ICU, the median number of PaCO_2_ measurements per patient was 43. The factors significantly associated with unfavorable neurological outcomes were age, Hunt and Kosnik grade, maximum lactate levels during the first 24 h, and maximum (odds ratio [OR], 1.12; 95% confidence interval [CI], 1.03–1.21; *p* < 0.01) and minimum PaCO_2_ levels (OR, 0.81; 95% CI, 0.72–0.92; *p* < 0.01). Receiver operating characteristic curve analysis revealed that the cutoff range of PaCO_2_ was 30.2–48.3 mmHg. Unfavorable neurological outcomes were noted in 78.8% of patients with PaCO_2_ levels outside this range and in 22.8% of patients with PaCO_2_ levels within this range.

**Conclusions:**

Both the maximum and minimum PaCO_2_ levels during ICU management in patients with SAH were significantly associated with unfavorable neurological outcomes. Further prospective studies are required to validate our findings and explore their clinical implications. Our findings may provide a scientific rationale for these future prospective studies.

**Electronic supplementary material:**

The online version of this article (10.1186/s40560-018-0353-1) contains supplementary material, which is available to authorized users.

## Background

Aneurysmal subarachnoid hemorrhage (SAH) accounts for a reasonably large proportion of stroke-related mortality cases. The overall mortality in patients with SAH is over 30%, and approximately 10–20% of survivors show functional dependence despite intensive neurological care [1, 2]. Several extensive studies have been conducted to improve intensive neurological care in patients with SAH [3–8].

Cerebral blood flow (CBF) is mainly regulated by arterial carbon dioxide tension (PaCO_2_) [9, 10]. Abnormal PaCO_2_ levels are considered to cause major changes in CBF through vasoconstriction and vasodilation, respectively, possibly contributing to further brain injury [11, 12]. Additionally, a previous systematic review demonstrated abnormal PaCO_2_ levels to be associated with poor clinical outcomes in patients with traumatic brain injury (TBI), post-cardiac arrest patients, and stroke patients [13]; therefore, PaCO_2_ control in the intensive care unit (ICU) can greatly influence the management of patients with SAH. Several studies have reported that hypocapnia (defined as PaCO_2_ < 35 mmHg), albeit unintentional, may be a common and under-recognized cause of brain tissue hypoxia after SAH and may be associated with poor neurological outcomes and a high incidence of delayed cerebral ischemia (DCI) [14–16]. However, the association between hypercapnia (defined as PaCO_2_ > 45 mmHg) and neurological outcomes in patients with SAH has not been examined.

The present guidelines for the management of SAH do not specify the target PaCO_2_ level [17, 18]. Recently, a phase I study involving poor-grade SAH patients demonstrated that CBF increased after controlled hypercapnia by up to 60 mmHg, even during periods of vasospasm [10]. Another study involving resuscitated cardiac arrest patients reported that the levels of neuron-specific enolase were lower with targeted therapeutic mild hypercapnia (PaCO_2_, 50–55 mmHg) than with normocapnia (PaCO_2_, 35–45 mmHg) [19]. Therefore, it is unknown whether hypercapnia is permitted and whether there is an optimal target PaCO_2_ level in patients with SAH.

The present study aimed to examine the association of abnormal PaCO_2_ levels with unfavorable neurological outcomes and investigate the target PaCO_2_ level in patients with SAH for further prospective studies.

## Materials and methods

### Study design and setting

This single-center, retrospective, observational study was performed at Kagawa University Hospital, a 613-bed teaching institution with an 8-bed ICU managed by a neurointensivist. The patient medical records were reviewed after receiving approval from the institutional review board (approval number H29-090), and the study was performed in accordance with the ethical standards established in the 1964 Declaration of Helsinki and its later amendments. The institutional review board approved a waiver of the requirement for individual consent. If patients did not want to participate in the current study, they could request to be excluded.

### Study participants and inclusion criteria

**Fig. 1:**
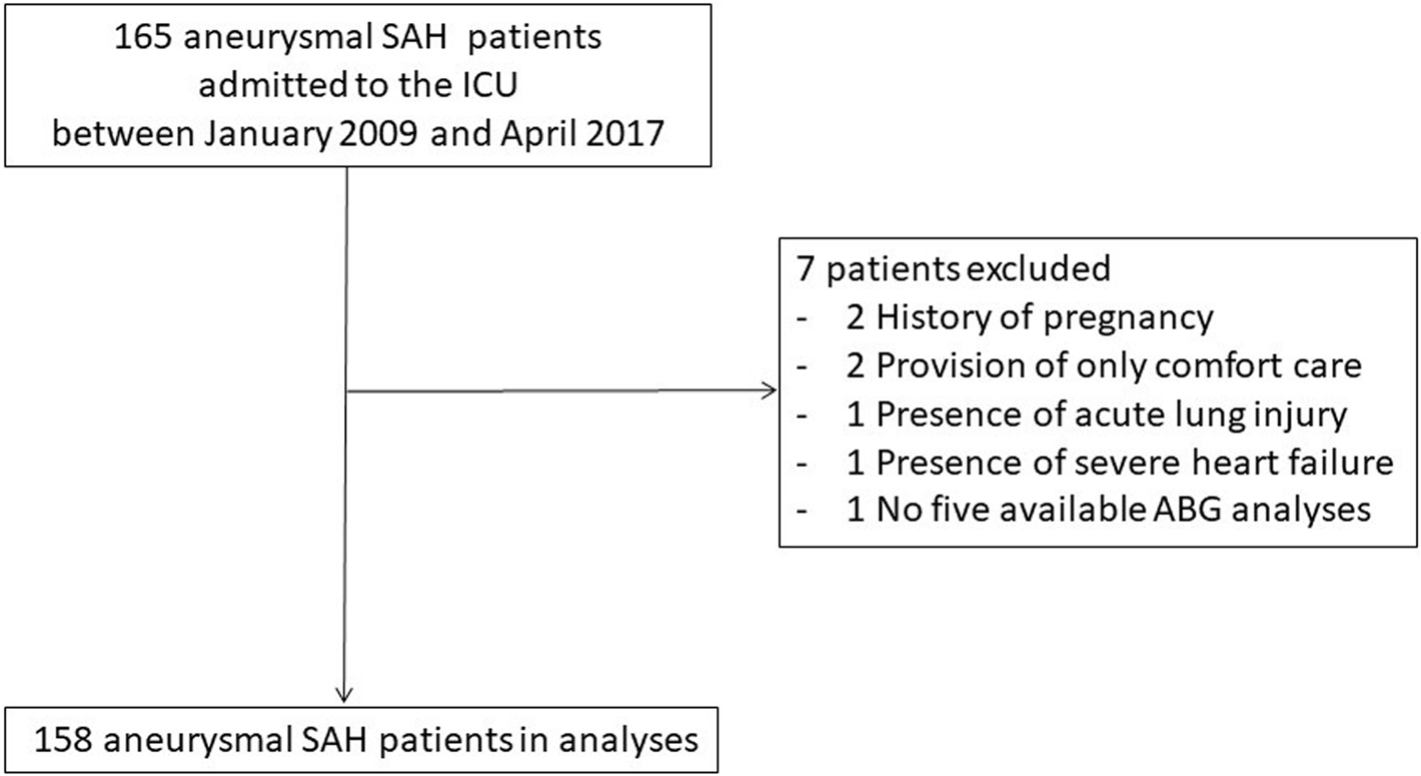
Study flow diagram. ABG indicates arterial blood gas

The study included patients aged > 18 years who were consecutively admitted to the ICU with a confirmed diagnosis of SAH between January 1, 2009, and April 15, 2017, and who had at least five available arterial blood gas (ABG) analyses. The exclusion criteria were a history of pregnancy or trauma, presence of acute lung injury/acute respiratory distress syndrome or severe heart failure requiring positive-pressure ventilation during the first 2 weeks in the ICU, and provision of only comfort care (Fig. 1).

### General management of SAH in the ICU

All the patients with SAH were managed in accordance with the Guidelines for the Management of Aneurysmal Subarachnoid Hemorrhage by the American Heart Association/American Stroke Association [8, 18]. In addition to general intensive care, all the patients were monitored for clinical deterioration or for the development of cerebral infarction owing to DCI. Using previously published criteria, DCI was defined as either (1) the development of new focal neurological signs or deterioration of the consciousness level thought to be caused by the presence of ischemia after the exclusion of other possible causes or (2) the appearance of new infarcts caused by vasospasm on CT or MRI [15, 20]. Fluid management was targeted to maintain euvolemia. Induced hypertension and hemodilution were not performed, and mannitol or hypertonic saline solution was not administered. The patients received minimum amounts of sedatives, such as propofol, midazolam, and dexmedetomidine, which were necessary to prevent ventilator dyssynchrony and patient discomfort. Analgesics, including acetaminophen, nonsteroidal anti-inflammatory medications, and fentanyl, were administered as required. Fever was treated aggressively with acetaminophen, nonsteroidal anti-inflammatory medications, or cooling devices. Active maintenance of normothermia was not routinely performed.

### Control of PaCO_2_ levels in patients with SAH in the ICU

ABG analyses, including PaCO_2_, were routinely performed every 6 h during the first 2 weeks in the ICU, and additional measurements were obtained by critical care physicians as needed. Patients were typically managed in the ICU during the first 2 weeks with reference to ABG analyses data, considering the timing of DCI occurrence. For patients in whom the arterial line could be withdrawn and who could be discharged from the ICU at an early stage, periodic ABG monitoring was completed by day 14. All patients were intubated prior to angiography and the initial treatment (surgical clipping or endovascular coiling) using a muscle relaxant. The muscle relaxant was discontinued after the completion of the initial treatment and was not used as ICU management thereafter. In mechanically ventilated patients, respiratory management usually involved the use of the spontaneous breathing mode (pressure support mode) with minimal support (i.e., continuous positive airway pressure or a pressure support level ≤ 5 cm H_2_O). Daily spontaneous breathing trials were not routinely performed. When a patient’s condition stabilized, he/she was usually weaned off mechanical ventilation and extubated after the completion of the initial treatment.

### Data sampling

The following data were collected: age, sex, Hunt and Kosnik (H&K) grade, treatment modality (coil or clip), number of PaCO_2_ measurements, PaCO_2_ levels, maximum and minimum PaCO_2_ levels during the first 2 weeks in the ICU, serum lactate levels, use of mechanical ventilation, modified Rankin scale (mRS) score at hospital discharge, DCI rate, mechanical ventilation duration, ICU stay duration, hospital stay duration, and hospital mortality.

### Outcome measures

The primary outcome was the association of abnormal PaCO_2_ with unfavorable neurological outcomes assessed using the mRS at hospital discharge [21]. The mRS is a measure of global disability and comprises the following seven outcome categories: no symptoms at all, no significant disability, slight disability, moderate disability, moderately severe disability, severe disability, and death. All the patients with SAH were evaluated in real time using the mRS at hospital discharge, and the findings were mentioned in their medical records. The neurological outcome was considered unfavorable when the mRS score was 3–6 and was considered favorable when the mRS score was 0–2. The secondary outcome was the association of abnormal PaCO_2_ with the presence of DCI.

### Statistical analysis

The patients were divided into two groups according to their neurological outcomes (unfavorable and favorable outcome groups). Descriptive statistics are used to summarize the demographic factors and baseline characteristics. The groups were compared using Student’s *t* test or the Mann–Whitney *U* test, as deemed appropriate. Fisher’s exact test was used to make the categorical comparisons. Alterations in the PaCO_2_ levels were examined separately for each clinical grade and SAH outcome. Univariate and multivariate analyses were performed to determine the independent predictors of unfavorable neurological outcomes. The covariates of age (> 65 years), sex (female), H&K grade, treatment modality (coil or clip), maximum lactate levels during the first 24 h [4, 8, 22], and maximum and minimum PaCO_2_ levels during the first 2 weeks in the ICU were included in the multivariate analysis based on published reports [23, 24]. The Mann–Whitney *U* test was used to estimate the receiver operating characteristic (ROC) curve and area under the curve (AUC). For each continuous variable, the cutoff value that had the best combination of sensitivity and specificity was identified. The patients were subdivided further into four groups according to the maximum and minimum PaCO_2_ levels during the first 2 weeks in the ICU. The statistical analyses were performed using EZR (Saitama Medical Center, Jichi Medical University, Saitama, Japan), a graphical user interface for R (The R Foundation for Statistical Computing, Vienna, Austria); more precisely, it is a modified version of R commander designed to add statistical functions that are frequently used in biostatistics [25]. A two-sided *p* value < 0.05 was considered statistically significant in all the analyses. Missing data were not replaced or estimated.

## Results

### Baseline characteristics of the study population

The study included 158 patients (mean age, 62.9 years; 52 men; Table 1). The median number of PaCO_2_ measurements per patient during the first 2 weeks in the ICU was 43. Unfavorable neurological outcomes were observed in 46.2% of the patients. Table 1 presents the comparisons of clinical characteristics between the unfavorable and favorable outcome groups. On univariate analysis, the groups significantly differed with regard to age (*p* < 0.01), H&K grade (*p* < 0.01), SD of PaCO_2_ levels (*p* < 0.01), and maximum (*p* < 0.01) and minimum PaCO_2_ levels (*p* < 0.01) during the first 2 weeks in the ICU. On comparing the unfavorable and favorable outcome groups, it was found that the unfavorable outcome group had a significantly longer median (interquartile range) mechanical ventilation duration (9 [2–16] days vs. 1 [1, 2] days, *p* < 0.01), ICU stay duration (17 [13–21] days vs. 14 [12–16] days, *p* < 0.01), and hospital stay duration (41 [26–74] days vs. 24 [21–30] days, *p* < 0.01).

**Table 1:** Characteristics of the study population

Variables	All patients (*N* = 158)	Unfavorable outcome group (*N* = 73)	Favorable outcome group (*N* = 85)	*p* value
Age (years)	62.9 ± 15.9	69.6 ± 15.1	57.1 ± 14.4	< 0.01
Sex (male)	52 (32.9)	23 (31.5)	29 (34.1)	0.74
H&K grade				< 0.01
I	13 (8.2)	3 (4.1)	10 (11.8)	
II	60 (38.0)	14 (19.2)	46 (54.1)	
III	41 (25.9)	22 (30.1)	19 (22.4)	
IV	29 (18.4)	23 (31.5)	6 (7.1)	
V	15 (9.5)	11 (15.1)	4 (4.7)	
Treatment modality				0.27
Coil	117 (74.1)	58 (79.5)	59 (69.4)	
Clip	39 (24.7)	14 (19.2)	25 (29.4)	
Number of arterial blood gas analyses on days 1–14	43 [31–55]	53 [39–56]	40 [28–48]	< 0.01
Median PaCO_2_ on days 1–14 (mmHg)	39.0 [37.2–40.9]	38.4 [36.9–40.5]	39.4 [37.4–40.9]	0.11
SD of PaCO_2_ on days 1–14	3.63 [2.75–4.60]	4.05 [3.58–5.20]	2.97 [2.45–3.95]	< 0.01
Proportion of PaCO_2_ ≤ 30 among all blood gas analyses on days 1–14 (%)	0 [0–2]	2 [0–5]	0 [0–0]	< 0.01
Proportion of PaCO_2_ < 35 among all blood gas analyses on days 1–14 (%)	14 [3–26]	21 [9–35]	9 [2–22]	< 0.01
Proportion of PaCO_2_ > 45 among all blood gas analyses on days 1–14 (%)	4 [0–16]	7 [2–18]	2 [0–12]	< 0.05
Maximum PaCO_2_ level on days 1–14 (mmHg)	47.4 [43.7–51.0]	49.4 [45.6–51.9]	45.8 [42.9–49.8]	< 0.01
Minimum PaCO_2_ level on days 1–14 (mmHg)	31.0 [28.7–33.8]	29.4 [26.2–32.7]	31.7 [30.2–34.5]	< 0.01
Use of mechanical ventilation	156 (98.7)	73 (100)	83 (97.6)	0.50
Modified Rankin scale score				< 0.01
0	29 (18.4)	0 (0.0)	29 (34.1)	
1	27 (17.1)	0 (0.0)	27 (31.8)	
2	29 (18.4)	0 (0.0)	29 (34.1)	
3	24 (15.2)	24 (32.9)	0 (0.0)	
4	27 (17.1)	27 (37.0)	0 (0.0)	
5	12 (7.6)	12 (16.4)	0 (0.0)	
6	10 (6.3)	10 (13.7)	0 (0.0)	
DCI	23 (14.6)	16 (21.9)	7 (8.2)	< 0.05
Mechanical ventilation duration	2 [1–9]	9 [2–16]	1 [1–2]	< 0.01
ICU stay duration	15 [12–19]	17 [13–21]	14 [12–16]	< 0.01
Hospital stay duration	28 [22–51]	41 [26–74]	24 [21–30]	< 0.01
Hospital mortality	10 (6.3)	10 (13.7)	0 (0.0)	< 0.01

Data are presented as mean ± standard deviation (SD), number (percentage), or median [interquartile range]

*H&K* Hunt and Kosnik, *DCI* Delayed cerebral ischemia, *ICU* Intensive care unit

No significant differences in median PaCO_2_ levels were observed between the unfavorable and favorable outcome groups (*p* = 0.11); however, the unfavorable outcome group had significantly higher proportions of hypocapnia (PaCO_2_ < 35 mmHg; *p* < 0.01) and hypercapnia (PaCO_2_ > 45 mmHg; *p* = 0.02) among all the blood gas analyses during the first 2 weeks in the ICU. The episodes of hypocapnia were not intentional, and most involved an associated pH level of > 7.45 and not < 7.35. The relationships between the neurological outcomes of each SAH severity (H&K grades) and PaCO_2_ are presented in Additional file 1: Table S1. Although there was no significant difference in the median of PaCO_2_ or proportion of hypocapnia and hypercapnia across H&K grades, there were significant differences in minimum PaCO_2_ levels and outcomes across H&K grades.

### Alterations in PaCO_2_ levels during the first 2 weeks in the ICU according to neurological outcomes

**Fig. 2:**
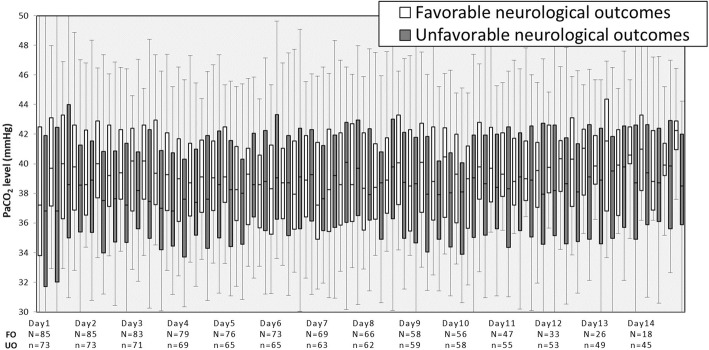
Alterations in PaCO_2_ levels during the first 2 weeks in the intensive care unit according to neurological outcomes. The boxes represent the 25th to 75th percentile. The whiskers represent the 5th to 95th percentile. UO, unfavorable neurological outcomes; FO, favorable neurological outcomes

As Fig. 2 shows, during the early stage of hospitalization (days 1–5), PaCO_2_ levels were lower in the unfavorable outcome group than in the favorable outcome group. Subsequently, about 1 week after hospitalization (days 6–8), almost no differences in PaCO_2_ levels were found between the groups. However, in the late stage of hospitalization (days 9–14), PaCO_2_ levels were again lower in the unfavorable outcome group than in the favorable outcome group.

### Alterations in PaCO_2_ levels during the first 2 weeks in the ICU according to disease severity (H&K grade)

**Fig. 3:**
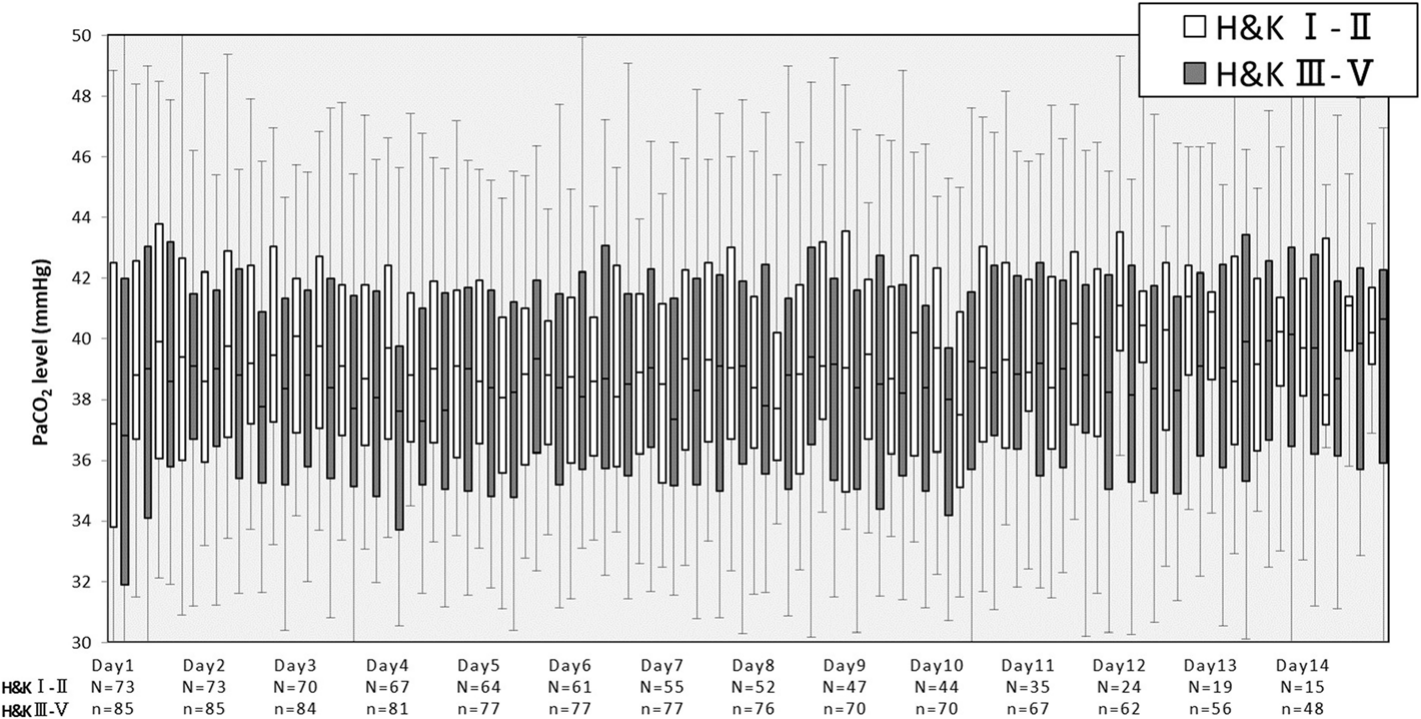
Alterations in PaCO_2_ levels during the first 2 weeks in the intensive care unit according to the Hunt and Kosnik grade of subarachnoid hemorrhage. The boxes represent the 25th to 75th percentile. The whiskers represent the 5th to 95th percentile

As Fig. 3 shows, during the early stage of hospitalization (days 1–5), PaCO_2_ levels were lower in patients with moderate-to-severe disease grade (H&K grades III–V) than in those with mild disease grade (H&K grades I–II). However, after day 6, almost no differences in PaCO_2_ levels were found between the patient groups.

### Predictors of unfavorable neurological outcomes in patients with SAH

On multiple regression analysis (Table 2), unfavorable neurological outcomes were significantly associated with age, H&K grade, maximum lactate levels during the first 24 h, and maximum (odds ratio [OR], 1.12; 95% confidence interval [CI], 1.03–1.21; *p* < 0.01) and minimum PaCO_2_ levels (OR, 0.81; 95% CI, 0.72–0.92; *p* < 0.01).

**Table 2:** Multivariate analysis of factors that influenced unfavorable outcomes

Variables	OR	95% CI	*p* value
Age > 65 years	7.62	2.97–19.60	< 0.01
Sex (female)	1.01	0.38–2.67	0.98
H&K grade	2.43	1.53–3.87	< 0.01
Treatment modality (clip)	0.69	0.27–1.75	0.43
Maximum lactate levels during the first 24 h (mmol/L)	1.34	1.02–1.76	< 0.05
Maximum PaCO_2_ level (mmHg)	1.11	1.03–1.21	< 0.01
Minimum PaCO_2_ level (mmHg)	0.82	0.73–0.93	< 0.01

*OR* odds ratio; *CI* confidence interval, *H&K* Hunt and Kosnik

### ROC curve analysis

ROC curves of unfavorable neurological outcomes according to the maximum and minimum PaCO_2_ levels in the ICU were constructed. Table 3 presents the respective AUCs, sensitivities, and specificities for predicting unfavorable neurological outcomes. The optimal cutoff values for the maximum and minimum PaCO_2_ levels were 48.3 and 30.2 mmHg, respectively, during the first 2 weeks in the ICU.

**Table 3:** Receiver operating characteristic curve analysis of maximum and minimum PaCO_2_ levels

Variables	AUC	95% CI	Optimal cutoff	Sensitivity	Specificity
Maximum PaCO_2_ level	0.641	0.55–0.73	48.3 mmHg	58.9	68.2
Minimum PaCO_2_ level	0.696	0.61–0.78	30.2 mmHg	60.3	74.1

*AUC* area under the curve, *CI* confidence interval

### Association between PaCO_2_ levels and unfavorable neurological outcomes

**Fig. 4:**
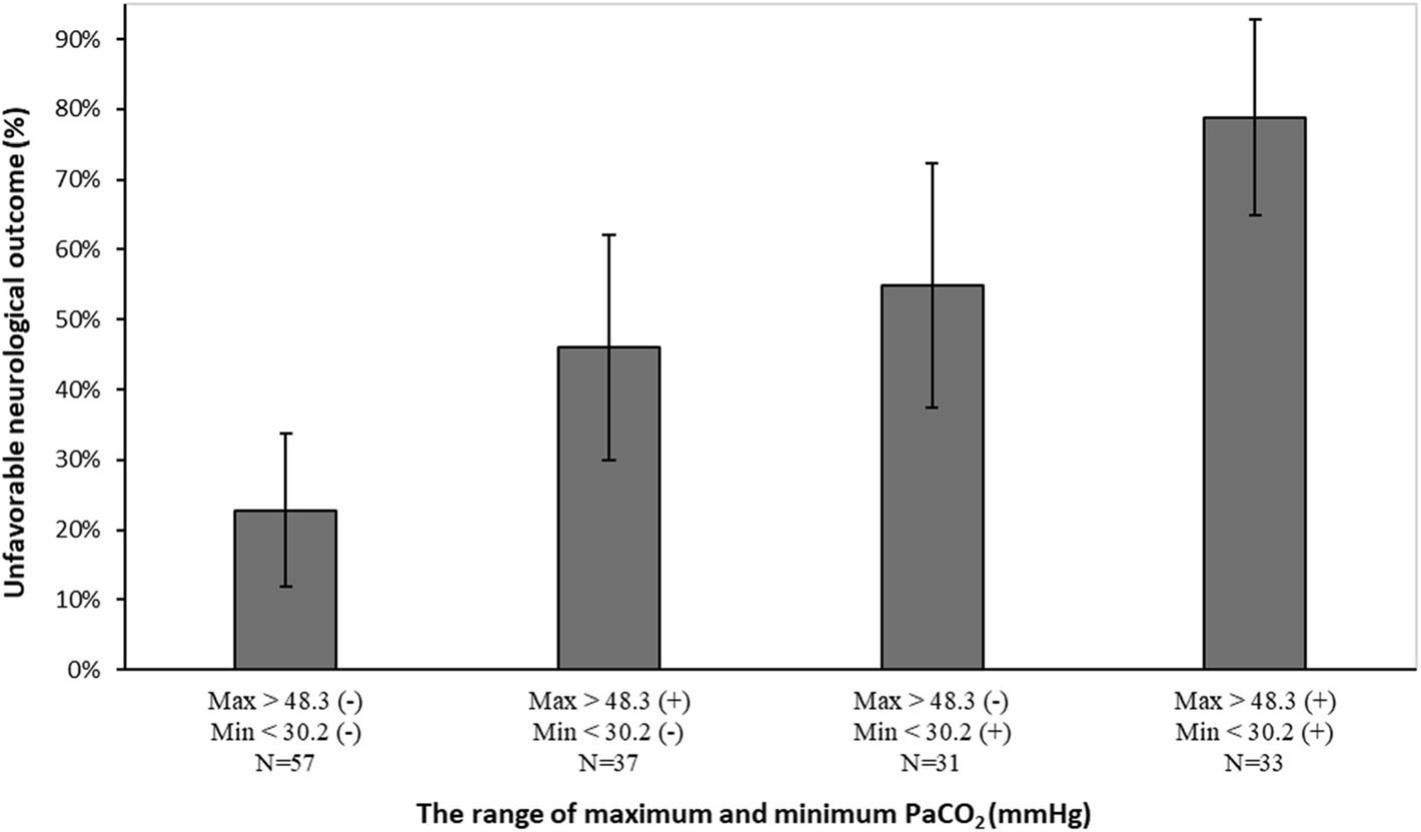
Association between PaCO_2_ levels and unfavorable neurological outcomes. Error bars represent 95% confidence intervals (CIs)

We found that 78.8% of patients with a maximum PaCO_2_ of > 48.3 mmHg and a minimum PaCO_2_ of < 30.2 mmHg had unfavorable neurological outcomes, whereas only 22.8% of patients who did not have either of these PaCO_2_ levels had unfavorable neurological outcomes (Fig. 4).

### Association between PaCO_2_ levels and DCI

DCI was observed in 14.6% of patients. The median period from admission to the occurrence of DCI was 8 (range, 7–9) days. The presence of DCI was confirmed during the first 2 weeks in the ICU. Additional file 1: Table S2 presents comparisons of the clinical characteristics between patients with DCI and those without DCI. On univariate analysis, there were no significant differences in age, sex, treatment modality, median PaCO_2_ levels, and proportions of hypocapnia and hypercapnia between patients with DCI and those without DCI, although the number of patients with DCI was relatively small. On the other hand, the maximum PaCO_2_ level, SD of PaCO_2_, and ICU and hospital stay durations were significantly greater in patients with DCI than in those without DCI. In addition, DCI was associated with unfavorable neurological outcomes. On multiple regression analysis (Additional file 1: Table S3), DCI was significantly associated with the maximum PaCO_2_ level (OR, 1.1; 95% CI, 1.02–1.19; *p* < 0.05).

### Alterations in PaCO_2_ levels during the first 2 weeks in the ICU according to the presence/absence of DCI

**Fig. 5:**
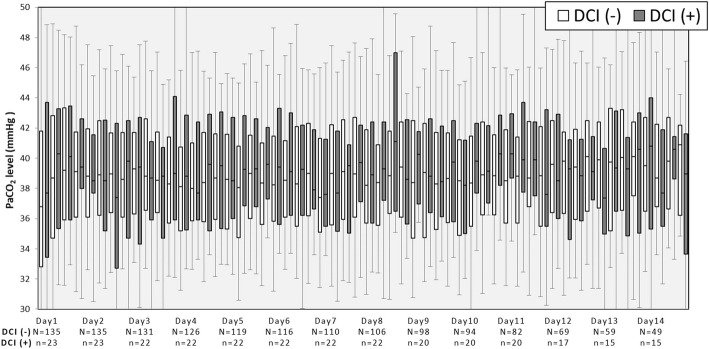
Alterations in PaCO_2_ levels during the first 2 weeks in the intensive care unit according to presence/absence of DCI. The boxes represent the 25th to 75th percentile. The whiskers represent the 5th to 95th percentile

As Fig. 5 shows, although the number of patients with DCI was relatively small, patients with DCI showed increased PaCO_2_ variability during the first 2 weeks in the ICU when compared with the findings in patients without DCI.

## Discussion

In the present study, we demonstrated that both the maximum and minimum PaCO_2_ levels during ICU management were significantly associated with unfavorable neurological outcomes in patients with SAH. Moreover, we investigated the thresholds for both hypocapnia and hypercapnia to examine the target PaCO_2_ level during ICU management in patients with SAH, which might help in further prospective studies with adjustment for factors, such as respiratory settings and sedation.

### Association between hypocapnia and neurological outcomes

Several previous studies reported that hypocapnia was associated with poor neurological outcomes or DCI [14, 15]. Williamson et al. reported that severe hypocapnia, which was defined as PaCO_2_ ≤ 30 mmHg, was significantly associated with poor neurological outcomes (OR, 4.52; *p* < 0.01) [15], and this finding is consistent with our result. However, they reported that moderate hypocapnia, which was defined as PaCO_2_ < 35 mmHg, was not independently associated with poor neurological outcomes (OR, 1.30; *p* = 0.58) [15]. Hypocapnia was previously shown to be associated with brain tissue hypoxia and poor outcomes in patients with TBI [26, 27]. Therefore, the current guidelines do not support the use of prophylactic and induced hypocapnia (PaCO_2_ < 30 mmHg) [28]. Consistent with these findings and guidelines, avoidance of hypocapnia, especially a PaCO_2_ level of < 30 mmHg, is presumed to be desirable in the management of patients with SAH.

### Association between hypercapnia and neurological outcomes

As shown in Fig. 2, on days 1–14, the 75th percentile of PaCO_2_ levels appeared to not be significantly different between the unfavorable and favorable outcome groups. This result indicated that some patients with unfavorable outcomes had higher PaCO_2_ levels with greater variation when compared with the findings in those with favorable outcomes. It remains unknown whether a hypercapnia strategy to avoid hypocapnia in patients with SAH is permitted. The association between hypercapnia and poor outcomes has been suggested to be secondary to hypercapnia- induced cerebral vasodilation, increased intracranial pressure, and decreased cerebral perfusion [13, 29, 30]. Alternatively, a previous report involving SAH patients with a poor clinical grade (Hunt/Hess III–V) investigated the association between controlled hypercapnia and CBF or brain tissue oxygen saturation (StiO_2_) [10]. The report mentioned that although controlled hypercapnia of up to 60 mmHg reproducibly increased CBF and StiO_2_, the PaCO_2_ reactivity of CBF, which was calculated by dividing the change in CBF by the change in PaCO_2_, was the highest at PaCO_2_ levels between 40 and 50 mmHg and was slightly decreased at higher PaCO_2_ levels. Impaired PaCO_2_ reactivity has been reported to be frequent after SAH, particularly in patients with a poor clinical grade, and associated with DCI [31]. Considering our current data and the importance of PaCO_2_ reactivity and possibly the cerebral steal phenomenon, hypercapnia of up to approximately 50 mmHg might be permitted as a therapeutic strategy. However, there are limited studies, and thus, further investigations are necessary.

### Association between PaCO_2_ levels and DCI

Several previous studies reported that hypocapnia was associated with DCI [14, 15]. In our study, a small number of patients had DCI, and no significant association was noted between DCI and hypocapnia. According to our current data, hypercapnia or PaCO_2_ variability might have been associated with DCI. However, further studies are necessary.

### Mechanism of abnormal PaCO_2_ levels

The mechanism of the association between abnormal PaCO_2_ levels and neurological outcomes is unknown, and it was difficult to discuss about the causal relationship of abnormal PaCO_2_ levels owing to the retrospective nature of our study as well as previous studies and the absence of adjustment for factors, such as respiratory settings and sedation [13]. However, severe brain injury following SAH itself might directly induce abnormal PaCO_2_ levels [15]. In the present study, we found that 22.8% of patients (13 patients) with PaCO_2_ levels within the cutoff range (30.2–48.3 mmHg) had unfavorable neurological outcomes. In these 13 patients, the mean age was relatively high (70.4 ± 11.4 years) and 69.2% (9 patients) had H&K grades III–V. We concluded that these factors, besides the abnormal PaCO_2_ levels, could also affect the outcomes. Meanwhile, unfavorable neurological outcomes were noted in 78.8% of the patients with PaCO_2_ levels outside the cutoff range (26 patients). For these patients, we also determined the duration during which their PaCO_2_ levels were outside the range. ABG analyses represent PaCO_2_ level at a certain time point; we assumed that PaCO_2_ level among blood gas samples was linear. Although there was no significant difference among the neurological outcomes, the duration outside the cutoff range tended to be longer in the 26 patients with unfavorable neurological outcomes compared with the 7 patients with favorable neurological outcomes (33.8 ± 15.6 h vs. 25.1 ± 9.7 h, *p* = 0.17). Regarding this point, we concluded that a significant difference could have been noted if the number of patients was large. Accordingly, further detailed study regarding the influence of the duration during which the PaCO_2_ levels are outside the range on neurological outcomes seems to be necessary.

### Clinical implementation

The findings of the present study collectively suggested that both hypocapnia and hypercapnia were associated with unfavorable neurological outcomes after SAH. Our study design was not controlled intentionally, and a causal relationship was unclear. Our results should be regarded as hypothesis generating. Therefore, further prospective studies with adjustment for factors such as respiratory settings and sedation are necessary to determine a more appropriate PaCO_2_ target (30–40 or 40–50 mmHg) and to evaluate the causal relationship of abnormal PaCO_2_ levels and the effectiveness of end-tidal CO_2_ monitoring in CO_2_ management. Moreover, our findings might indicate the issue of PaCO_2_ variability, and PaCO_2_ can fluctuate greatly similar to glucose [3, 32, 33]. Thus, the intentional control of PaCO_2_ might be beneficial, especially in the first 72 h after hemorrhage when early brain injury occurs [8].

### Limitations

The present study had several limitations. First, this was a retrospective observational study conducted at a single center, and thus, there was potential selection bias. Moreover, uncontrolled confounding factors may have been present. Second, the neurological outcomes of patients after discharge were not assessed. Third, the possible impact of differences in the patient population, such as those brought about by comorbidities, on neurological outcomes could not be definitively excluded. Fourth, the vital signs, sedation dose, oxygen dose, end-tidal CO_2_ level, and specific mechanical ventilator settings, including the minute volume, fraction of inspired oxygen, and positive end-expiratory pressure during each ABG analysis, were not sufficiently considered. Therefore, it was difficult to assess the causal relationship of abnormal PaCO_2_ levels and examine whether the PaCO_2_ levels obtained in this study were intentionally controlled. Fifth, the study sample size was relatively small. Sixth, as this study had a small number of patients with DCI, the association between DCI and outcomes was not sufficiently analyzed. Finally, as the current study examined the limited association between abnormal PaCO_2_ levels and neurological outcomes in patients with SAH, the effect of the regulation of abnormal PaCO_2_ levels was not determined. Furthermore, as details of the causes of abnormal PaCO_2_ levels, such as inadequate use of sedatives, antipyretic agents, or muscle relaxants, and the oxygen concentration or pressure support during mechanical ventilation were not obtained owing to the retrospective nature of this study, we could not make definitive suggestions on the management of PaCO_2_ in a clinical setting. The results of our study should be regarded as hypothesis generating. Therefore, further prospective studies, including randomized controlled trials, are required to confirm the effects of abnormal PaCO_2_ levels on neurological outcomes in patients with SAH.

## Conclusions

We found that hypocapnia and hypercapnia during ICU management in patients with SAH were significantly associated with unfavorable neurological outcomes. Further prospective studies are required to validate our findings and explore their clinical implications. Our findings may provide a scientific rationale for these future prospective studies.

## Additional file

Additional file 1: **Table S1.** Baseline characteristics of the study population across H&K grades. **Table S2.** Association between PaCO_2_ levels and DCI (univariate analysis). **Table S3.** Multivariate analysis of factors that influenced DCI. (DOCX 36 kb)

## Abbreviations

ABG: Arterial blood gas
CBF: Cerebral blood flow
CI: Confidence interval
DCI: Delayed cerebral ischemia
H&K: Hunt and Kosnik
ICU: Intensive care unit
OR: Odds ratio
PaCO_2_: Arterial carbon dioxide tension
SAH: Subarachnoid hemorrhage
TBI: Traumatic brain injury
