# Supplementary material for: Association of abnormal carbon dioxide levels with poor neurological outcomes in aneurysmal subarachnoid hemorrhage: a retrospective observational study
Source: J Intensive Care. 2018 Dec 17;6:83. doi: 10.1186/s40560-018-0353-1 (PMC6296027; doi:10.1186/s40560-018-0353-1)
Supplement: Supplementary file 1 — Table S1. Baseline characteristics of the study population across H&K grades. Table S2. Association between PaCO2 levels and DCI (univariate analysis). Table S3. Multivariate analysis of factors that influenced DCI. (DOCX 36 kb) [file 40560_2018_353_MOESM1_ESM.docx]

Table S1. Baseline characteristics of the study population across H&K grades

| Variables | H&K  grade I  (N = 13) | H&K  grade II  (N = 60) | H&K  grade III  (N = 41) | H&K  grade IV  (N = 29) | H&K  grade V  (N = 15) | *p*-value |
| --- | --- | --- | --- | --- | --- | --- |
| Age (years) | 63.6 ± 17.4 | 63.2 ± 15.7 | 60.6 ± 16.2 | 65.3 ± 14.8 | 62.8 ± 18.4 | 0.82 |
| Sex (male) | 4 (30.8) | 14 (23.3) | 16 (39.0) | 9 (31.0) | 9 (60.0) | 0.09 |
| Median PaCO_2_ on days 1–14 (mmHg) | 39.8  [36.3–41.1] | 39.2  [37.5–40.8] | 38.3  [36.4–40.7] | 38.6  [37.3–40.4] | 37.7  [36.1–41.2] | 0.77 |
| Proportion of PaCO_2_ < 35 among all ABG analyses on days 1–14 (%) | 12 [5–26] | 9 [3–22] | 20 [6–26] | 14 [5–30] | 22 [8–44] | 0.19 |
| Proportion of PaCO_2_ > 45 among all ABG analyses on days 1–14 (%) | 6 [0–20] | 3 [0–14] | 7 [0–15] | 3 [0–16] | 7 [1–21] | 0.66 |
| Maximum PaCO_2_ level on days 1–14 (mmHg) | 47.1  [42.8–49.8] | 46.3  [43.4–50.3] | 48.4  [44.6–51.9] | 46.3  [44.3–49.9] | 50.2  [45.3–57.3] | 0.06 |
| Minimum PaCO_2_ level on days 1–14 (mmHg) | 32.9  [30.8–34.0] | 31.7  [29.4–34.5] | 29.8  [27.7–31.8] | 29.7  [26.5–33.9] | 30.9  [29.4–32.9] | <0.05 |
| Unfavorable outcome  (mRS 3–6) | 3 (23.1) | 14 (23.3) | 22 (53.7) | 23 (79.3) | 11 (73.3) | <0.01 |
| DCI | 0 (0.0) | 4 (6.7) | 13 (31.7) | 5 (17.2) | 1 (6.7) | <0.01 |
| Mechanical ventilation duration | 1 [1–1] | 1 [1–2] | 2 [1–11] | 9 [2–17] | 10 [5–14] | <0.01 |
| ICU stay duration | 11 [8–14] | 14 [12–16] | 16 [13–20] | 19 [16–22] | 15 [12–19] | <0.01 |
| Hospital stay duration | 22 [19–26] | 25 [21–33] | 32 [24–59] | 45 [35–81] | 32 [16–57] | <0.01 |
| Hospital mortality | 0 (0.0) | 1 (1.7) | 3 (7.3) | 2 (6.9) | 4 (26.7) | <0.05 |

Data are expressed as mean ± standard deviation, number (percentage), or median [interquartile range].

H&K, Hunt and Kosnik; ABG, arterial blood gas; mRS, modified Rankin scale score; DCI, delayed cerebral ischemia; ICU, intensive care unit.

Table S2. Association between PaCO_2_ levels and DCI (univariate analysis)

| Variables | All patients  (N = 158) | DCI  (N = 23) | No DCI  (N = 135) | *p*-value |
| --- | --- | --- | --- | --- |
| Age (years) | 62.9 ± 15.9 | 62.9 ± 14.5 | 62.9 ± 16.2 | 0.99 |
| Sex (male) | 52 (32.9) | 8 (34.8) | 44 (32.6) | 0.82 |
| H&K grades |  |  |  | <0.01 |
| I | 13 (8.2) | 0 (0.0) | 13 (9.6) |  |
| II | 60 (38.0) | 4 (17.4) | 56 (41.5) |  |
| III | 41 (25.9) | 13 (56.5) | 28 (20.7) |  |
| IV | 29 (18.4) | 5 (21.7) | 24 (17.8) |  |
| V | 15 (9.5) | 1 (4.3) | 14 (10.4) |  |
| Treatment modality |  |  |  | 0.10 |
| Coil | 117 (74.1) | 14 (60.9) | 103 (76.3) |  |
| Clip | 39 (24.7) | 8 (34.8) | 31 (23.0) |  |
| Number of arterial blood gas analyses on days 1–14 | 43 [31–55] | 53 [40–56] | 42 [30–54] | <0.05 |
| Median PaCO_2_ on days 1–14 (mmHg) | 39.0 [37.2–40.9] | 39.5 [37.7–40.7] | 38.6 [37.0–40.9] | 0.48 |
| SD of PaCO_2_ on days 1–14 | 3.63 [2.75–4.60] | 4.03 [3.63–5.33] | 3.45 [2.71–4.30] | <0.01 |
| Proportion of PaCO_2_ ≤ 30 among all blood gas analyses on days 1–14 (%) | 0 [0–2] | 0 [0–3] | 0 [0–2] | 0.76 |
| Proportion of PaCO_2_ < 35 among all blood gas analyses on days 1–14 (%) | 14 [3–26] | 20 [6–26] | 13 [3–26] | 0.60 |
| Proportion of PaCO_2_ > 45 among all blood gas analyses on days 1–14 (%) | 4 [0–16] | 7 [3–19] | 4 [0–15] | 0.10 |
| Maximum PaCO_2_ level on days 1–14 (mmHg) | 47.4 [43.7–51.0] | 51.0 [46.4–55.0] | 46.9 [43.5–50.3] | <0.01 |
| Minimum PaCO_2_ level on days 1–14 (mmHg) | 31.0 [28.7–33.8] | 30.4 [28.2–33.7] | 31.0 [28.7–33.8] | 0.43 |
| Modified Rankin scale score |  |  |  | <0.05 |
| 0 | 29 (18.4) | 1 (4.3) | 28 (20.7) |  |
| 1 | 27 (17.1) | 1 (4.3) | 26 (19.3) |  |
| 2 | 29 (18.4) | 5 (21.7) | 24 (17.8) |  |
| 3 | 24 (15.2) | 9 (39.1) | 15 (11.1) |  |
| 4 | 27 (17.1) | 3 (13.0) | 24 (17.8) |  |
| 5 | 12 (7.6) | 2 (8.7) | 10 (7.4) |  |
| 6 | 10 (6.3) | 2 (8.7) | 8 (5.9) |  |
| Mechanical ventilation duration | 2 [1–9] | 3 [1–13] | 2 [1–9] | 0.55 |
| ICU stay duration | 15 [12–19] | 20 [16–22] | 14 [12–18] | <0.01 |
| Hospital stay duration | 28 [22–51] | 38 [30–77] | 26 [22–44] | <0.01 |
| Hospital mortality | 10 (6.3) | 2 (8.7) | 8 (5.9) | 0.64 |

Data are presented as mean ± standard deviation, number (percentage), or median [interquartile range].

H&K, Hunt and Kosnik; DCI, delayed cerebral ischemia; ICU, intensive care unit

Table S3. Multivariate analysis of factors that influenced DCI

| Variables | OR | 95% CI | *p*-value |
| --- | --- | --- | --- |
| Age > 65 years | 0.62 | 0.24–1.59 | 0.32 |
| H&K grade | 1.23 | 0.82–1.85 | 0.32 |
| Maximum PaCO_2_ level (mmHg) | 1.10 | 1.02–1.19 | <0.05 |

OR, odds ratio; CI, confidence interval; H&K, Hunt and Kosnik
